# Girls behind Counters

**Published:** 1875-02

**Authors:** 


### GIRLS BEHIND COUNTERS.

It is literally a barbarity to require shop-girls to stand behind counters all day, waiting on customers; if they are in motion, or in actual attendance on customers, no appreciable harm may result, because the varying motions divide the strain among many muscles, but if standing still, the whole strain is on one set, more especially so upon the ligaments or bands which support the distinctive organ of the sex, causing "falling," or other misplacement, which set up debilities and drains which often make all after life a burden, and many times a failure. The moment there is no customer there should be permission to take a seat. And yet the imperative rule in many stores is to compel a standing position from morning till night; this is an inexcusable cruelty, and no humane and intelligent person will countenance the practice after having learned the facts.

